# Trends and characteristics of terrorist attacks using edged weapons over five decades: implications for mass casualty preparedness

**DOI:** 10.3389/fpubh.2026.1887631

**Published:** 2026-08-04

**Authors:** Fayez Alruqi

**Affiliations:** Emergency Medical Services Program, Department of Nursing, College of Nursing and Health Sciences, Jazan University, Jazan, Saudi Arabia

**Keywords:** counter-terrorism medicine, disaster medicine, edged weapons, mass casualty preparedness, terrorism

## Abstract

**Introduction:**

Terrorist attacks using edged weapons have increased globally over the past decade. Their epidemiology over a sustained period remains poorly characterized, limiting the evidence available to inform mass casualty preparedness. This study aimed to describe the global distribution of these attacks over five decades, quantify their kill-to-wound ratio (KWR) relative to firearm and explosive attacks, and characterize attack, target, and perpetrator patterns.

**Methods:**

A retrospective cross-sectional study was conducted using the Global Terrorism Database, covering 1970 to 2020. Attacks were identified using a precoded primary weapon classification. Events flagged as doubtful terrorism were excluded. The KWR was calculated using complete-case casualty data and a Poisson-based method with 95% confidence intervals (CI) and compared with firearm and explosive comparator datasets extracted using the same filters.

**Results:**

A total of 2,616 eligible attacks were identified across 12 regions and 120 countries. Two temporal surges were identified: 1989 to 1997, peaking at 177 attacks, and 2014 to 2020, accounting for 43.7% of all attacks and peaking at 235 attacks in 2015. The complete-case aggregate KWR was 2.45 (95% CI 2.35–2.55), exceeding that of firearms (1.76, 95% CI 1.74–1.78) and explosives (0.38, 95% CI 0.37–0.38), with non-overlapping confidence intervals across all three weapon categories. The edged weapon KWR was 1.39 times that of firearms and 6.45 times that of explosives. Of 109 identified lone-actor attacks, 85.3% occurred between 2014 and 2020, concentrated in Western Europe (47.7%) and North America (27.5%); however, lone actors accounted for 8.1% of all attacks during that period (93 of 1,144). Lone-actor attacks produced a KWR of 0.34 compared with 2.67 for group-actor attacks. The median casualty count was one victim per attack.

**Conclusion:**

Edged weapon terrorism produced an aggregate fatality-dominant casualty profile that differed from the wound-dominant pattern of explosive attacks. Preparedness frameworks informed primarily by blast and ballistic injury may not fully reflect this profile. Context-specific planning should account for both the aggregate fatality-dominant pattern and the potential wound-dominant profile seen in coordinated multi-perpetrator events.

## 1 Introduction

Terrorism remains a persistent threat to public safety worldwide. Over the past five decades, more than 200,000 terrorist incidents have been recorded globally (1). Weapon-specific patterns within this burden remain unevenly described. Mass casualty preparedness has largely been informed by explosive and firearm attacks, which account for the majority of terrorism-related fatalities (2). However, attacks involving edged weapons have increased in recent years across multiple regions (3). Edged weapons are widely accessible and require minimal technical expertise, planning, or logistical support (4). Despite these operational concerns, the global epidemiology of edged weapon terrorism remains poorly understood.

Injury patterns in edged weapon attacks differ from those observed after explosive attacks (5). Stab and slash wounds commonly involve anatomically vulnerable regions, including the neck, chest, and upper abdomen (6). Whereas explosive attacks typically generate larger numbers of wounded survivors, edged weapon attacks may produce a higher proportion of fatalities relative to wounded casualties, with implications for triage models calibrated to blast injury (2). However, coordinated multi-perpetrator events, such as the 2014 Kunming Railway Station attack, may generate a high proportion of surviving wounded requiring immediate hemorrhage control and damage control resuscitation (7, 8).

Existing studies on edged weapon attacks are either geographically limited or do not isolate edged weapons from broader weapon categories (5, 9). Weapon-specific analyses using terrorism databases have been conducted for other attack types, including chemical incidents, (10) and attacks on specific target settings (11). However, no equivalent global analysis has examined edged weapon terrorism across a sustained historical period. As a result, its temporal distribution, geographical spread, casualty burden, and variation across lone-actor incidents, coordinated assaults, and mass casualty events remain insufficiently characterized. This limits the evidence available to inform mass casualty preparedness for this attack modality.

This study used retrospective global terrorism data to characterize the epidemiology of terrorist edged weapon attacks over five decades. The primary aim was to describe their global temporal and geographical distribution. Secondary objectives were to quantify the kill-to-wound ratio (KWR) and compare it with that of firearm and explosive attacks; describe attack type, target type, and perpetrator characteristics; and identify high-casualty events within the dataset.

## 2 Materials and methods

### 2.1 Study design

This study used a retrospective cross-sectional descriptive design to examine the global epidemiology of terrorist edged weapon attacks between 1970 and 2020. This design has been widely adopted in counter-terrorism medicine research to characterize the frequency and casualty patterns of specific attack types. Reporting follows the Strengthening the Reporting of Observational Studies in Epidemiology (STROBE) statement for cross-sectional studies (12), and a completed checklist is provided as a Supplementary file.

### 2.2 Data source

Data were obtained from the Global Terrorism Database (GTD), an open-source event-level database maintained by the National Consortium for the Study of Terrorism and Responses to Terrorism (START) at the University of Maryland (13). The GTD contains records of more than 200,000 terrorist incidents worldwide, spanning from 1 January 1970 to 31 December 2020. Entries are compiled from publicly available materials including media reports, news archives, books, journals, and legal documents. Each incident must be documented by at least one high-quality source. Four organizations have been responsible for GTD data collection at different periods: the Pinkerton Global Intelligence Service (PGIS) covered 1970 to 1997, the Center for Terrorism and Intelligence Studies (CETIS) covered 1998 to March 2008, the Institute for the Study of Violent Groups (ISVG) covered April 2008 to October 2011, and START staff covered November 2011 onwards. The GTD codebook cautions that shifts in recorded frequencies around these transitions may partly reflect methodological changes rather than actual trends in terrorism. This is acknowledged in the interpretation of temporal patterns throughout this study.

### 2.3 GTD definition of terrorism

The GTD defines a terrorist incident according to three core inclusion criteria and three supplementary criteria. For an incident to be included, it must be intentional, involve violence or the immediate threat of violence, and be carried out by non-state actors. In addition, at least two of the following three criteria must be met: the act must be conducted in pursuit of a political, economic, religious, or social objective; it must be intended to coerce, intimidate, or convey a message to an audience beyond the immediate victims; and it must fall outside the boundaries of legitimate warfare under international humanitarian law.

### 2.4 Inclusion criteria

All GTD entries from 1 January 1970 to 31 December 2020 were considered. Attacks were identified by filtering for entries classified as “Knife or Other Sharp Object” in the pre-coded primary weapon subtype field, covering knives, machetes, pangas, cleavers, axes, swords, and daggers. Two previously separate categories, “Knife” and “Sharp Object Other Than Knife,” were retroactively merged into this classification and applied uniformly across the database. Attacks where an edged weapon was recorded only as a secondary or tertiary weapon were outside the scope of this analysis and were not separately identified or counted.

### 2.5 Exclusion criteria

After applying the primary weapon subtype filter, attacks flagged as doubtful terrorism were excluded. Incidents classified as confirmed terrorism were retained. Incidents with missing classification status were also retained; a sensitivity analysis restricted to confirmed terrorism incidents only is reported separately. This criterion did not exclude all civil-conflict-era incidents. Numerous attacks recorded during the Rwandan genocide and the Algerian Civil War were classified as confirmed terrorism and remained in the primary analysis. Their influence was examined separately in the geographical sensitivity analysis.

### 2.6 Variables

Variables were selected for their clinical and epidemiological relevance and for their consistent availability across the full study period. The year and month of the attack were used as temporal variables. Region, country, and city described the geographical spread. Attack type classified the nature of each event. Whether an attack was successful and whether it involved a suicide element were also recorded. Target type and target subtype described the victims. Perpetrator group name identified the responsible organization, and the lone-actor variable flagged attacks carried out by unaffiliated individuals with no known group affiliation. The lone-actor variable was systematically coded for incidents from 1998 onwards. Three pre-1998 incidents carried this flag in the GTD source data, reflecting retrospective coding during database revisions; these were retained in the analysis, yielding a total of 109 lone-actor incidents. It excludes cases where group affiliation was suspected but unconfirmed, leading to systematic undercounting. The specific weapon used was extracted from the GTD free-text weapon detail field and classified into categories using keyword matching. This field was available for a subset of attacks only. Fatalities and non-fatal injuries captured the casualty burden. Both include recorded perpetrator casualties, and these could not be consistently separated from civilian or responder casualties in the GTD records. Attack setting was classified using the vicinity variable. A value of 0 indicates an attack within city boundaries, and a value of 1 indicates an attack in the immediate vicinity of a city, following GTD coding. These are reported as urban and peri-urban, respectively, for ease of reporting, representing an operational relabeling rather than a strict demographic classification. Lone-actor attacks were additionally stratified by era, region, and primary target type.

### 2.7 Missing data

Fatality data were missing for 26 attacks, and injury data were missing for 126 attacks. These values were not imputed. For total victim count per attack, killed and wounded values were summed where available. When one casualty component was missing, the total victim count represented the available recorded component only and was therefore interpreted as a minimum recorded casualty burden. Missing values are concentrated in regions with limited media coverage and in the earlier collection period, making the mechanism unlikely to be random. All casualty totals are presented with explicit denominators. The KWR was calculated where both fatality and injury data were available. Attacks with zero non-fatal injuries alongside confirmed fatalities were retained, as zero wounded is a plausible outcome in targeted assassinations. However, some zero values may reflect unreported injuries. Because wounded counts were missing in some fatality-producing events, the complete-case ratio may underestimate or overestimate the true ratio. The direction of bias cannot be determined from GTD records alone. Two attacks carried unresolved setting codes and could not be classified as urban or peri-urban; these were reported as unknown for attack setting.

### 2.8 Statistical analysis

Statistical analyses were performed using R version 4.2.2 (R Foundation for Statistical Computing, Vienna, Austria). The following R packages were used: readxl for data import, dplyr for data manipulation, ggplot2 for data visualization, and tidyr for data reshaping. Categorical variables were presented as frequencies with percentages. Casualty variables were reported as totals with denominators. Precision estimates for KWRs were calculated using a Poisson-based method. Confidence intervals (CI) were derived from the log-ratio, with standard errors computed as the square root of the sum of the reciprocals of the killed and wounded counts, consistent with comparable analyses in counter-terrorism medicine. The KWR was calculated as the aggregate total killed divided by the aggregate total wounded across all complete-case attacks.

For descriptive purposes, a surge year was defined as any year in which the annual attack count exceeded the dataset mean plus one standard deviation. This rule was specified before data examination, although the resulting threshold was necessarily computed from the data (mean = 53.4; SD = 59.7; threshold = 113.1 attacks per year). Surge periods were defined as broader clusters of elevated annual counts surrounding years that exceeded the threshold. Boundaries were assigned to contiguous or near-contiguous periods in which annual counts remained above the long-run mean, allowing isolated intervening years below the threshold. This approach was used to describe temporal patterning rather than to infer formal change-points. Formal change-point analysis was considered. The Pruned Exact Linear Time (PELT) algorithm with a Bayesian Information Criterion (BIC) penalty identified 37 change-points across the annual time series, yielding an over-specified solution that was not interpretable in a descriptive context. Casualty distribution per attack was characterized using mean, median, and interquartile range across the full included dataset.

### 2.9 Sensitivity analysis

Two pre-specified sensitivity analyses were conducted. The first excluded attacks from Sub-Saharan Africa and Algeria, where the majority of high-casualty events are attributable to the Rwandan genocide and the Algerian Civil War. This geographical exclusion is the primary mechanism for distinguishing civil-conflict-era mass atrocity events from ideologically motivated terrorism. The second sensitivity analysis excluded events with fatality counts at or above the 99th percentile, defined by the observed threshold of 50 or more confirmed fatalities. All events sharing the threshold value were excluded. This event-level approach provides an alternative to geographical exclusion and avoids removing lower-casualty events from the same regions.

Firearm and explosive comparator datasets were extracted using the same analytic filters as the edged-weapon dataset: the primary weapon field only, with events flagged as doubtful terrorism excluded. This filter retained both confirmed terrorism events and events with missing classification status, consistent with the primary edged weapon analysis. Firearm attacks were identified using the GTD weapon type classification “Firearms” and explosive attacks using the classification “Explosives.” Edged-weapon attacks were identified at the weapon subtype level using the classification “Knife or Other Sharp Object,” because the primary weapon type field groups all melee weapons together without distinguishing knives from other instruments. KWRs were calculated using consistent complete-case denominators across all three weapon categories.

### 2.10 Ethical considerations

This study used publicly available, de-identified data from the GTD. No primary data collection involving human participants was conducted, and no identifiable or clinical information was accessed. Formal ethical review was not required.

## 3 Results

### 3.1 Overall dataset

**Figure 1:**
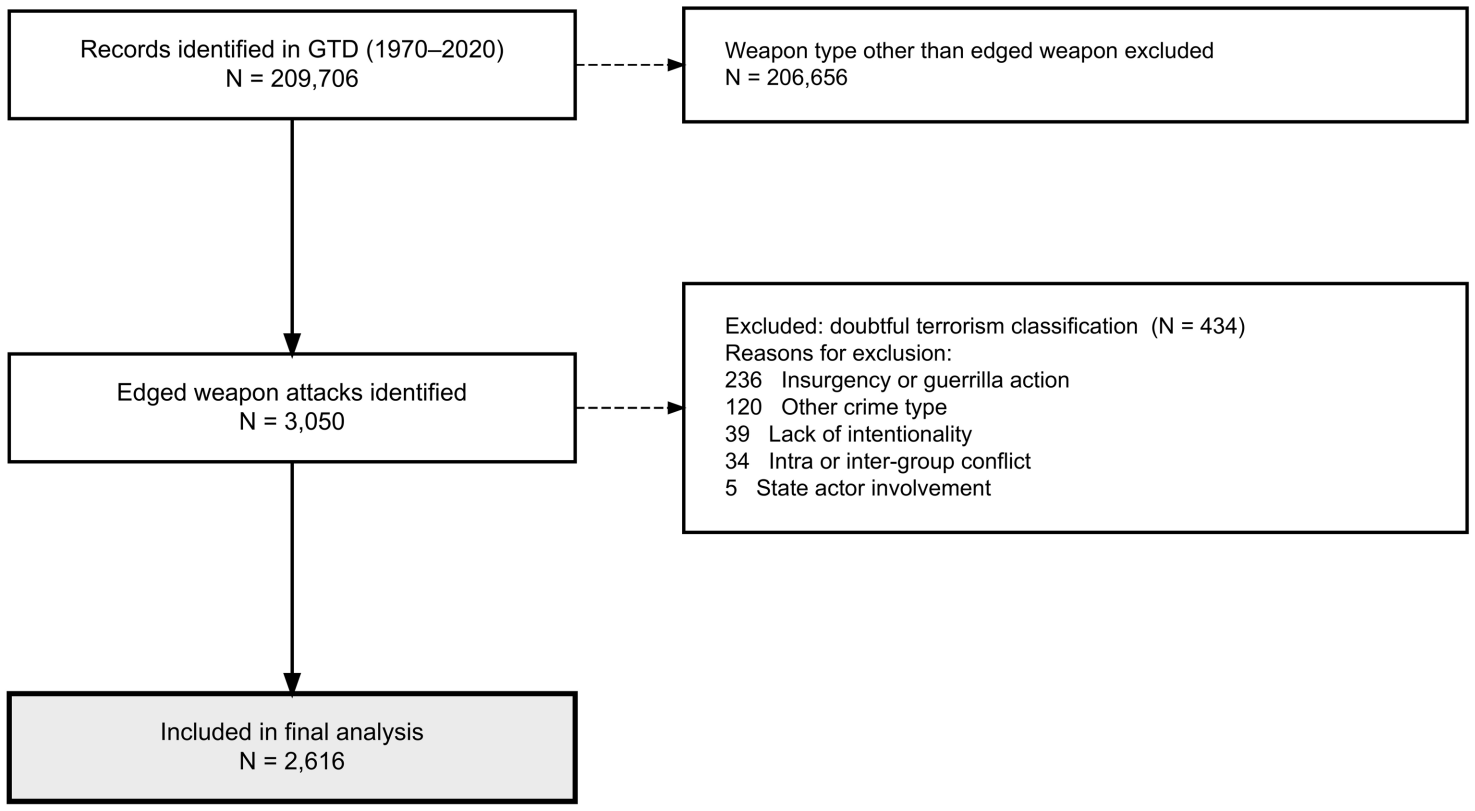
Inclusion and exclusion flow diagram. GTD, Global Terrorism Database.

A total of 209,706 incidents were recorded in the GTD from 1970 to 2020. Following application of inclusion and exclusion criteria, 2,616 eligible terrorist edged weapon attacks were included (Figure 1).

The characteristics of all attacks are summarized in Table 1. Attacks were identified across all 12 regions and 120 countries. The Middle East and North Africa were the most affected region (*n* = 901, 34.4%), followed by South Asia (*n* = 679, 26.0%) and Sub-Saharan Africa (*n* = 479, 18.3%). These three regions accounted for 78.7% of all attacks. Armed assault was the predominant attack type (*n* = 1,390, 53.1%). Private citizens and property were the most frequently targeted group (*n* = 1,471, 56.2%). The overall success rate was 90.1% (*n* = 2,356). The perpetrator group was unknown in 804 attacks (30.7%). The most frequently identified perpetrator category was West Bank and Gaza-based groups, accounting for 326 attacks (12.5%). Suicide attacks were rare (*n* = 10, 0.4%). Attacks attributed to unaffiliated lone individuals were recorded in 109 incidents, representing 4.2% of the full dataset. Most attacks occurred within city boundaries (*n* = 2,345, 89.6%), with 269 (10.3%) in peri-urban settings. Attack setting was unknown in two attacks (0.1%).

**Table 1:** Characteristics of terrorist attacks involving edged weapons.

Characteristic	*N* (%) or value
Region	
Middle East and North Africa	901 (34.4%)
South Asia	679 (26.0%)
Sub-Saharan Africa	479 (18.3%)
Western Europe	164 (6.3%)
Southeast Asia	117 (4.5%)
South America	69 (2.6%)
East Asia	66 (2.5%)
North America	54 (2.1%)
Central America and Caribbean	38 (1.5%)
Eastern Europe	34 (1.3%)
Central Asia	8 (0.3%)
Australasia and Oceania	7 (0.3%)
Top 10 countries	
India	360 (13.8%)
West Bank and Gaza Strip	293 (11.2%)
Algeria	266 (10.2%)
Israel	145 (5.5%)
Nigeria	92 (3.5%)
Afghanistan	90 (3.4%)
Democratic Republic of the Congo	86 (3.3%)
Pakistan	73 (2.8%)
Bangladesh	71 (2.7%)
Somalia	69 (2.6%)
Attack type	
Armed assault	1,390 (53.1%)
Assassination	654 (25.0%)
Hostage taking (kidnapping)	512 (19.6%)
Hijacking	26 (1.0%)
Facility or infrastructure attack	16 (0.6%)
Hostage taking (barricade incident)	14 (0.5%)
Other or unknown	4 (0.2%)
Target type	
Private citizens and property	1,471 (56.2%)
Police	303 (11.6%)
Government (general)	220 (8.4%)
Business	111 (4.2%)
Religious figures or institutions	105 (4.0%)
Journalists and media	68 (2.6%)
Military	59 (2.3%)
Educational institution	55 (2.1%)
Transportation	40 (1.5%)
Government (diplomatic)	34 (1.3%)
Other	150 (5.7%)
Attack characteristics	
Successful attack	2,356 (90.1%)
Unsuccessful attack	260 (9.9%)
Suicide attack	10 (0.4%)
Lone-actor (unaffiliated individual)*	109 (4.2%)
Perpetrator group	
West Bank and Gaza-based groups	326 (12.5%)
Communist Party of India–Maoist (CPI-Maoist)	230 (8.8%)
Al-Shabaab	68 (2.6%)
Taliban	61 (2.3%)
Unknown perpetrator	804 (30.7%)
Victims	
Killed, total (*n* = 2,590 attacks with data)	10,695
Wounded, total (*n* = 2,490 attacks with data)	3,636
Killed, complete-case subset (*n* = 2,476)	8,690
Wounded, complete-case subset (*n* = 2,476)	3,550
Kill-to-wound ratio (*n* = 2,476 attacks with complete data)	2.45 (95% CI 2.35–2.55)

*The lone-actor variable was systematically coded from 1998 onwards. Three pre-1998 incidents carried this flag in the GTD source data and were retained descriptively.

### 3.2 Temporal trends

**Figure 2:**
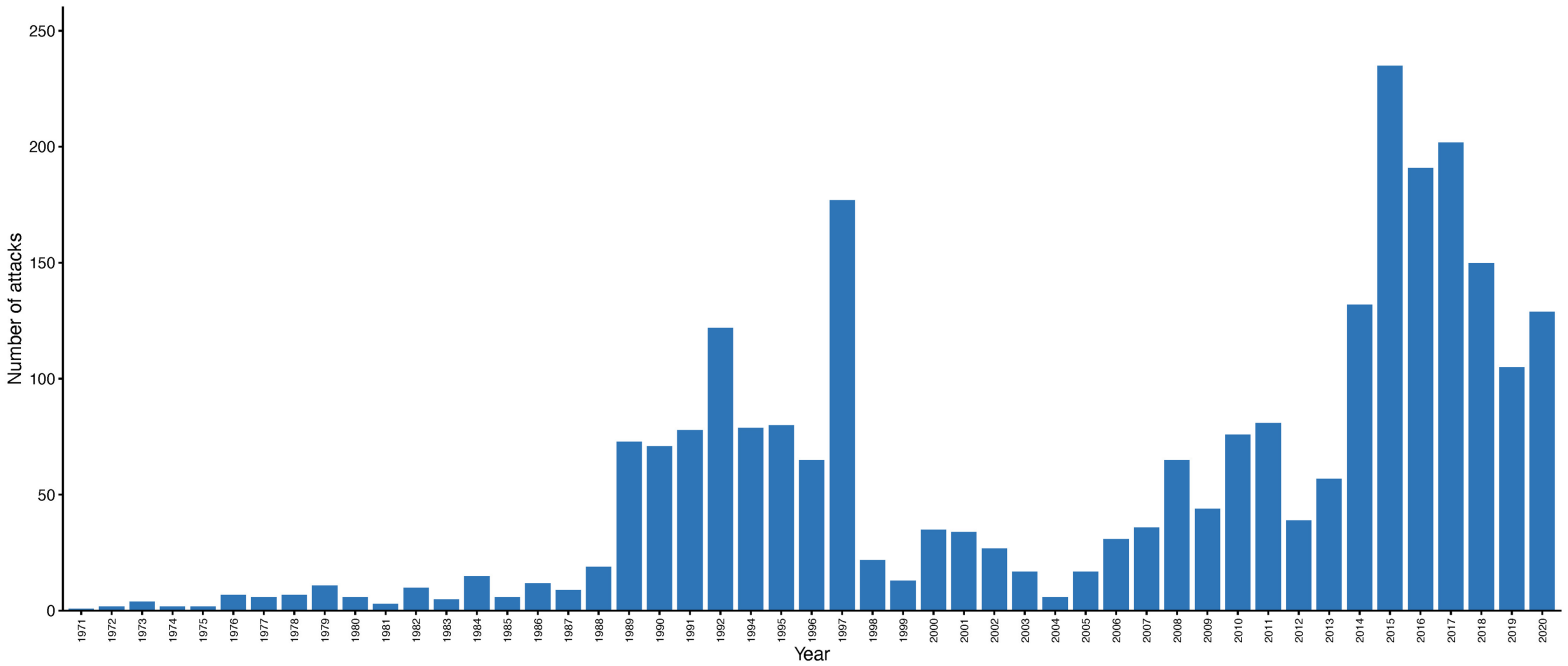
Number of eligible terrorist edged weapon attacks per year, 1971–2020 (*N* = 2,616).

The annual distribution of attacks is presented in Figure 2. Fewer than 20 attacks were recorded in any single year between 1971 and 1988. Two distinct temporal surges were identified using the pre-specified rule (any year exceeding the dataset mean plus one standard deviation; threshold = 113.1 attacks per year). The first surge spanned 1989 to 1997, during which 1992 and 1997 exceeded the threshold, with a peak of 177 attacks in 1997. Many attacks during this period occurred in Algeria and Sub-Saharan Africa, including events recorded during periods of civil conflict and mass violence. Annual counts then declined between 1998 and 2013, ranging from 6 to 81 with a mean of 38.4 per year. The second surge spanned 2014 to 2020, during which six years exceeded the threshold (2014, 2015, 2016, 2017, 2018, and 2020), with a peak of 235 attacks in 2015, the highest single-year count in the full 50-year period. In 2019, the number of attacks fell below the threshold at 105 but remained substantially above the long-run mean of 53.4 attacks per year. Three years (2015, 2016, 2017) also exceeded the mean plus two standard deviations (172.8 attacks per year). The 2014–2020 period accounted for 1,144 attacks (43.7% of the dataset). Monthly distribution was consistent across the calendar year. October recorded the highest count (*n* = 272, 10.4%) and April the lowest (*n* = 163, 6.2%).

### 3.3 Attack, target, and perpetrator characteristics

Attack type, target type, and perpetrator group distributions are summarized in Table 1. Specific weapon information was recorded in the GTD weapon detail field for 1,803 attacks (68.9%). Among these, knives were the most frequently identified weapon (*n* = 1,093, 60.6%), followed by sharp objects not further specified (*n* = 317, 17.6%), machetes (*n* = 161, 8.9%), multiple edged weapons (*n* = 84, 4.7%), and axes or hatchets (*n* = 78, 4.3%). Swords, daggers, saws, screwdrivers, and other named implements each accounted for less than 2%. The specific weapon was not recorded for 813 attacks (31.1%). A total of 109 attacks were identified as lone-actor incidents. By collection era: three attacks (2.8%) were recorded between 1971 and 1997; nine (8.3%) between 1998 and 2007; four (3.7%) between 2008 and 2013; and 93 (85.3%) between 2014 and 2020. Of the 1,144 attacks recorded during the second surge (2014–2020), lone-actor incidents accounted for 93 (8.1%). Lone-actor attacks were concentrated in Western Europe (*n* = 52, 47.7%) and North America (*n* = 30, 27.5%), accounting for 75.2% of all identified lone-actor incidents. Private citizens and property were the most common target type within lone-actor attacks (*n* = 46, 42.2%), followed by police (*n* = 15, 13.8%) and military targets (*n* = 9, 8.3%). Compared with group-actor attacks, lone-actor attacks were more frequently armed assaults (80.7% versus 51.9%), while group-actor attacks included higher proportions of assassination (25.6%) and hostage taking (20.3%).

### 3.4 Casualty distribution per attack

The distribution of casualties per attack was highly right-skewed. The median total victim count per attack was 1 (interquartile range [IQR] 1–3), and the mean was 5.48, reflecting a small number of high-casualty events driving the overall burden. A total of 166 attacks (6.3%) resulted in zero recorded casualties. The majority of attacks (*n* = 1,598, 61.1%) resulted in 1 to 2 total victims. Attacks resulting in 3 or more total victims numbered 852 (32.6%), those with 5 or more numbered 509 (19.5%), those with 10 or more numbered 272 (10.4%), and those with 25 or more numbered 99 (3.8%). The range across the full dataset was 0 to 766 victims per attack. The dataset comprises predominantly low-casualty events.

### 3.5 Casualty burden and kill-to-wound ratio

Among 2,590 attacks with fatality data, 10,695 persons were killed. Among 2,490 attacks with injury data, 3,636 were wounded. The KWR was calculated from the 2,476 attacks (94.6%) for which both values were available, yielding 8,690 killed and 3,550 wounded (KWR 2.45; 95% CI 2.35 to 2.55). The difference between the overall killed total (10,695) and the complete-case total (8,690) represents 2,005 fatalities in 114 attacks where killed was reported but wounded was not. Firearm attacks produced a KWR of 1.76 (95% CI 1.74 to 1.78) from 46,669 attacks with complete data. Explosive attacks produced a ratio of 0.38 (95% CI 0.37 to 0.38) from 84,521 attacks with complete data. The edged weapon KWR was 1.39 times that of firearms and 6.45 times that of explosives. Confidence intervals did not overlap between any pair of weapon categories.

**Figure 3:**
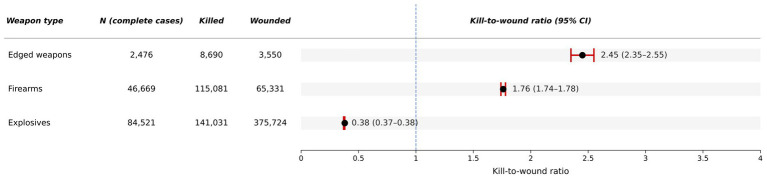
Kill-to-wound ratio by weapon type with 95% confidence intervals. Circle indicates point estimate. Horizontal lines indicate 95% confidence intervals. Dashed vertical line indicates a kill-to-wound ratio of 1.0 (equal kills and wounds). *N* reflects attacks with complete casualty data.

The comparison is presented in Figure 3. Stratified analyses revealed marked variation in the KWR by actor type, temporal period, and attack type. Lone-actor attacks produced a KWR of 0.34 (95% CI 0.27–0.42), substantially lower than group-actor attacks at 2.67 (95% CI 2.56–2.78). The first surge (1989–1997) had a KWR of 3.10 (95% CI 2.93–3.29) compared with 1.36 (95% CI 1.27–1.45) during the second surge (2014–2020). The 679 complete-case attacks falling outside both surge periods (1971–1988 and 1998–2013) produced a KWR of 3.27 (95% CI 2.99–3.57), the highest of the three temporal strata. Assassination had a higher KWR than armed assault (2.81, 95% CI 2.45–3.23 versus 2.23, 95% CI 2.14–2.33). Hostage taking produced the highest KWR of the three attack types at 5.50 (95% CI 4.76–6.35). Stratified KWR analyses for the three most frequent attack types are presented in Table 2 (Panel B); remaining attack type categories were not stratified due to small subgroup sizes.

**Table 2:** Kill-to-wound ratio: sensitivity and stratified analyses.

Analysis	*N* (complete cases)	Killed	Wounded	KWR	95% CI
Panel A: sensitivity analyses					
Primary analysis	2,476	8,690	3,550	2.45	2.35–2.55
Confirmed terrorism^*^	2,025	8,053	3,324	2.42	2.33–2.52
Excluding Sub-Saharan Africa and Algeria	1,803	2,907	2,106	1.38	1.31–1.46
Excluding top 1% of fatalities (≥50 killed)	2,457	6,627	2,917	2.27	2.18–2.37
Panel B: stratified kill-to-wound ratio analyses					
Lone-actor	109	113	336	0.34	0.27–0.42
Group-actor	2,367	8,577	3,214	2.67	2.56–2.78
First surge (1989–1997)	730	4,721	1,521	3.10	2.93–3.29
Non-surge periods (1971–1988 and 1998–2013)	679	2,078	636	3.27	2.99–3.57
Second surge (2014–2020)	1,067	1,891	1,393	1.36	1.27–1.45
Armed assault	1,315	6,661	2,983	2.23	2.14–2.33
Assassination	633	771	274	2.81	2.45–3.23
Hostage taking (kidnapping)	470	1,204	219	5.50	4.76–6.35

KWR, kill-to-wound ratio. *N*, number of attacks with complete casualty data (both killed and wounded recorded). 95% CI calculated using Poisson-based method. *Confirmed terrorism analysis restricted to incidents classified as confirmed terrorism (*n* = 2,148 attacks; complete cases *n* = 2,025), excluding the 468 incidents with missing classification status retained in the primary analysis.

### 3.6 Sensitivity analyses

**Figure 4:**
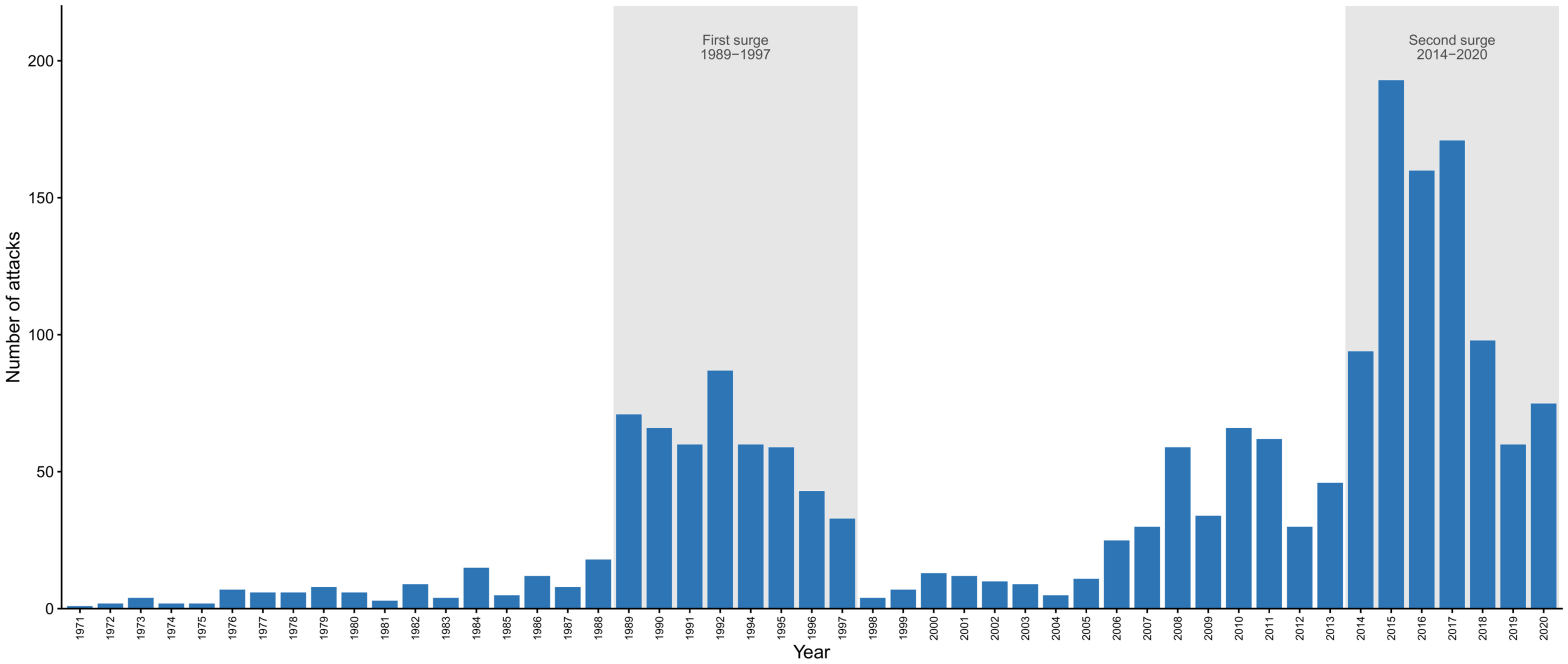
Number of eligible terrorist edged weapon attacks per year after exclusion of Sub-Saharan Africa and Algeria, 1971–2020 (*N* = 1,871). Shaded areas indicate identified surge periods (1989–1997 and 2014–2020).

Results of all sensitivity analyses are presented in Table 2. The top 1% fatality exclusion included 28 events with 50 or more confirmed fatalities. This exceeded the nominal 1% of the included dataset, approximately 26 of 2,616 attacks, because three events met the threshold of exactly 50 fatalities. The excluded events included the Ethiopia 2020 Mai Kadra incident, which reported 766 confirmed fatalities but had no data on wounded. As this event had no wounded data, it was already excluded from the complete-case KWR calculation. Because some excluded events lacked complete casualty data, the complete-case denominator decreased from 2,476 to 2,457. Excluding these 28 events produced a KWR of 2.27 (95% CI 2.18 to 2.37). The geographical sensitivity analysis excluding Sub-Saharan Africa and Algeria produced a KWR of 1.38 (95% CI 1.31 to 1.46) (Figure 4). Across both sensitivity analyses, the KWR remained above 1.0.

### 3.7 High-casualty events

The 20 attacks with the highest recorded victim burden are presented in Table 3. Of these, nine occurred in Sub-Saharan Africa and six occurred in Algeria. Together, these 15 events accounted for much of the civil-conflict era concentration that motivated the geographical sensitivity analysis. The highest recorded casualty event was the Mai Kadra massacre in Ethiopia in 2020, with 766 killed and wounded data not reported. The second and third highest casualty events occurred in Rwanda in 1997, with 271 killed and 227 wounded, and Algeria in 1997, with 256 killed and 194 wounded. The five highest casualty incidents accounted for 2,162 total recorded victims, representing 15.1% of the recorded casualty burden. Outside the civil conflict era concentration, the 2009 Urümqi incident had the highest recorded fatality count, although wounded data were not reported. Among complete-case events outside the civil-conflict-era concentration with both killed and wounded recorded, the largest was the Kunming Railway Station attack in China in 2014, with 33 killed and 143 wounded, yielding a KWR of 0.23. Four of the 20 highest casualty incidents had missing wounded data, and four recorded zero wounded despite high fatality counts.

**Table 3:** Twenty terrorist edged weapon attacks with the highest recorded victim burden.

Rank	Year	Country	Location	Killed	Wounded	Total	Perpetrator
1	2020	Ethiopia	Mai Kadra	766	NR	766	Samri militia
2	1997	Rwanda	Mutura	271	227	498	Hutu extremists
3	1997	Algeria	Sidi Rais	256	194	450	Unknown
4	1996	Burundi	Buhord	200	42	242	Tutsi extremists
5	1997	Algeria	Kherarba	206	0*	206	Unknown
6	1997	Algeria	Oued Rhiou	206	0*	206	Unknown
7	2009	China	Urümqi	184	NR	184	Unknown
8	2014	China	Kunming	33	143	176	Uighur Separatists
9	1997	Algeria	Baraki	85	67	152	Unknown
10	1992	India	Unknown	27	100	127	Muslim Militants
11	1997	Rwanda	Gisenyi	124	0*	124	Hutu extremists
12	1990	South Africa	Katlehong	21	100	121	Zulu Militants
13	1998	Burundi	Bandaguro district	120	NR	120	Unknown
14	1997	Algeria	Beni Messous	50	60	110	Unknown
15	2009	DR Congo	Busurungi	86	24	110	FDLR
16	2015	Nigeria	Gwoza	75	NR	75	Boko Haram
17	1992	Sri Lanka	Alinchipotana	56	17	73	LTTE
18	1996	Burundi	Gitega	70	2	72	Tutsi extremists
19	1984	Peru	Palca	21	45	66	Shining Path (SL)
20	1997	Algeria	Souhan district	64	0*	64	Unknown

Total victims = killed + wounded where both values were recorded. Where wounded was NR, total victims represent confirmed fatalities only. *Zero wounded reflect GTD source documentation and may indicate incomplete reporting. NR, not reported; FDLR, Democratic Forces for the Liberation of Rwanda; LTTE, Liberation Tigers of Tamil Eelam; SL, Sendero Luminoso; DR Congo, Democratic Republic of the Congo.

## 4 Discussion

This study provides a global descriptive analysis of terrorist edged weapon attacks over five decades using a precoded GTD weapon classification. The findings show that these attacks were geographically widespread but unevenly distributed, with the greatest burden recorded in the Middle East and North Africa, South Asia, and Sub-Saharan Africa. Although most incidents were low-casualty events, edged weapon attacks produced a substantially higher KWR than firearm and explosive attacks. A second surge was observed between 2014 and 2020, while a small number of high-casualty events, many linked to civil conflict-era settings, accounted for a large share of the recorded casualty burden.

The geographical distribution was also uneven. Although attacks were identified across all 12 regions and 120 countries, 78.7% occurred in three regions: the Middle East and North Africa, South Asia, and Sub-Saharan Africa. At the country level, India, the West Bank and Gaza Strip, and Algeria accounted for 35.2% of all included attacks. This concentration indicates that the global burden was shaped by both the first surge from 1989 to 1997, which included many events recorded in Algeria and Sub-Saharan Africa during periods of civil conflict and mass violence, and the second surge from 2014 to 2020. In contrast, Western Europe and North America together accounted for 8.4% of all attacks globally. However, they contributed 75.2% of all identified lone-actor incidents. This divergence is consistent with wider evidence that lone-actor terrorism has increased in high-income Western settings since 2010, with a concurrent shift toward low-technology attack methods including edged weapons (14, 15). These geographical findings should be interpreted alongside known variation in GTD source coverage and the sensitivity analyses excluding Sub-Saharan Africa and Algeria.

The KWR of 2.45 differs markedly from the 0.38 observed for explosive attacks. Edged weapon attacks produced fewer recorded wounded casualties relative to fatalities than explosive attacks, suggesting a different preparedness profile. Current mass casualty response frameworks and hemorrhage control initiatives, including those influenced by the Hartford Consensus, have often been shaped by firearm and explosive attack scenarios, where a high proportion of wounded patients may be expected (16, 17). Based on clinical literature on stabbing injuries, surviving casualties may present with penetrating vascular and thoracic injuries rather than the multiple-system blast injuries typical of explosive incidents (9). The 2014 Kunming Railway Station attack illustrates an exception. The coordinated, multi-perpetrator nature of the assault in an enclosed space was associated with a casualty profile in which wounded exceeded fatalities, as reported in the current study, more consistent with the explosive pattern. Lone-actor attacks and coordinated mass casualty incidents place different demands on pre-hospital resources, and triage protocols should account for both scenarios (18, 19).

The 2014 to 2020 period accounted for 43.7% of all attacks over the 50-year study period. However, lone-actor attacks accounted for 8.1% of all attacks during that period. They were concentrated in Western Europe (47.7%) and North America (27.5%) and most commonly targeted private citizens (42.2%) and police (13.8%). These findings are consistent with regional evidence from European settings suggesting a shift toward low-technology attack methods from 2014 onwards, though whether this pattern holds globally requires further investigation (20, 21). The lone-actor variable is available only after 1997 and likely underestimates the true proportion of unaffiliated perpetrators, particularly during the first surge period.

Perpetrator characteristics differed between lone-actor and group-actor attacks. Lone-actor attacks were concentrated in Western Europe and North America, occurred predominantly during the 2014–2020 surge, and were more frequently conducted as armed assaults against private citizens. By contrast, group-actor attacks accounted for most incidents globally. They exhibited a broader range of attack types, including assassination and hostage-taking, particularly in conflict-affected regions. Lone-actor attacks also produced a substantially lower KWR than group-actor attacks. These findings suggest that lone-actor and group-actor edged weapon attacks represent distinct operational patterns with different implications for preparedness and response. This geographical and operational distinction is consistent with previous studies describing the concentration of lone-actor terrorism in high-income Western settings (14, 15).

The median casualty count was one victim per attack (IQR 1 to 3), and 61.1% of attacks resulted in one to two total victims. Most edged weapon terrorist incidents in this dataset were low-casualty events, with a median of one victim per attack, suggesting that preparedness frameworks calibrated exclusively to mass casualty scenarios may not reflect the typical operational profile of these attacks. The high KWR and high-casualty outliers are important findings. However, the typical edged weapon attack in this dataset was a targeted, low-casualty event rather than a large-scale mass casualty incident.

Given the concentration of lone-actor attacks on private citizens in urban settings, early hemorrhage control before or shortly after emergency medical services (EMS) arrival may be important. Nevertheless, the GTD does not capture time to care or pre-hospital interventions. In a randomized trial of laypersons in a controlled setting, in-person bystander training achieved correct tourniquet application rates of approximately 88%, though skill retention at 3–9 months fell to around 55%, underscoring the need for refresher training programs (22). Police were a commonly targeted group in this dataset. In many settings, law enforcement may also be among the earliest responders. Tactical Emergency Casualty Care (TECC) training has been associated with effective on-scene stabilization of penetrating injuries (23). Expanding TECC training in jurisdictions with an elevated burden of lone-actor edged weapon attacks may be a reasonable preparedness strategy.

Hospital surge planning for edged weapon incidents should account for the lower proportion of recorded wounded relative to fatalities compared with explosive incidents. Based on clinical literature, surviving casualties from coordinated edged weapon events may present with penetrating vascular and thoracic injuries, placing demand on operating room capacity and blood product availability (24–26). The casualty distribution data in this study, which show that attacks resulting in 10 or more victims are uncommon (10.4% of all attacks), may help planners calibrate realistic surge scenarios rather than planning exclusively for worst-case events.

The use of the GTD precoded weapon classification provides a reproducible approach compared with previous analyses that relied on free-text identification or heterogeneous source databases (27). This approach is consistent with established counter-terrorism medicine research (11, 28, 29). Future research could apply these data to simulation modeling of pre-hospital response strategies, addressing the limitations of descriptive epidemiology in replicating real-world operational scenarios.

### 4.1 Limitations

The GTD is derived from publicly available sources, and completeness varies by region and time period. The database draws primarily on media and open-source reporting. Events in regions with limited media coverage or non-English reporting may be underrepresented. Sub-Saharan Africa and South Asia accounted for 44.3% of all included attacks. The true incidence in these regions may be underestimated because of variation in source availability and reporting completeness. Some high-casualty events are politically or historically contested and should be interpreted as GTD-classified events rather than definitive categorizations.

Classification and variable availability also varied across the study period. The end of the first surge in 1997 coincides precisely with the PGIS-to-CETIS collector transition, which the GTD codebook warns may cause frequency shifts independent of real-world changes; the first surge narrative should therefore be interpreted with caution. The second surge (2014–2020) falls entirely within the START collection period and is not subject to this confound. The lone-actor variable is available only after 1997 and may undercount lone-actor incidents during the first surge. The highest-casualty event, the Mai Kadra massacre in Ethiopia (2020), has contested attribution and no wounded data recorded; it does not affect the KWR but dominates the casualty distribution. The geographical sensitivity analysis excluded all attacks from Sub-Saharan Africa and Algeria, meaning lower-casualty events from these settings were also removed alongside civil-conflict-era events of interest. The alternative top 1% event-level analysis partially addressed this limitation. The temporal surge definition used a descriptive threshold rather than formal change-point inference.

Missing casualty data may also have affected the findings. Fatality and injury data were not imputed, and missing wounded data were concentrated partly in the high-casualty tail. The complete-case KWR may be affected, and the direction of bias cannot be determined. Casualty counts may also include perpetrator deaths or injuries where recorded. The GTD does not capture injury severity, anatomical injury distribution, time to care, pre-hospital interventions, hospital surge capacity, or the preventability of death. The findings provide indirect indicators of preparedness needs rather than direct measures of clinical outcomes. Incidence rates could not be estimated because the denominator was reported events rather than population exposure, and the GTD covers events only through December 2020.

## 5 Conclusion

This study provides a global characterization of terrorist edged weapon attacks over five decades using an established terrorism database. Edged weapon attacks produced an aggregate KWR of 2.45, substantially higher than both firearm and explosive attacks, and the ratio remained above 1.0 across sensitivity analyses. A second surge occurred between 2014 and 2020 and was accompanied by a concentration of identified lone-actor attacks in Western Europe and North America, although lone actors represented a minority of all attacks during this period. Most incidents involved one or two casualties, indicating that edged weapon terrorism should not be interpreted primarily through a mass casualty lens. These findings suggest that mass casualty preparedness frameworks informed by blast and ballistic injury patterns may not fully reflect the casualty profile of edged weapon terrorism. Context-specific planning should account for both the aggregate fatality-dominant profile observed in this dataset and the potential wound-dominant pattern seen in coordinated multi-perpetrator events.

## Data Availability

Publicly available datasets were analyzed in this study. This data can be found here: the data analyzed in this study were obtained from the Global Terrorism Database (GTD), maintained by the National Consortium for the Study of Terrorism and Responses to Terrorism (START) at the University of Maryland. Requests to access the dataset should be directed to START at: https://www.start.umd.edu/gtd.

## Supplementary material

Supplementary File 1 STROBE checklist for cross-sectional studies.
